# Editorial: Combining machine learning, computational modeling, and high throughput experimentation to accelerate discovery in systems microbiology

**DOI:** 10.3389/fmicb.2024.1451243

**Published:** 2024-07-17

**Authors:** Miguel Fuentes-Cabrera, Paolo Zuliani, Bowen Li, Jared L. Wilmoth, Aivett Bilbao, Alice C. Dohnalkova

**Affiliations:** ^1^Khoury College of Computer Sciences, Northeastern University, Oakland, CA, United States; ^2^Dipartimento di Informatica, Sapienza University of Rome, Rome, Italy; ^3^Research Software Engineering Group, Catalyst Building, Newcastle University, Newcastle upon Tyne, United Kingdom; ^4^Department of Environmental Science and Technology, University of Maryland, College Park, MD, United States; ^5^Environmental Molecular Sciences Laboratory, Pacific Northwest National Laboratory, Richland, WA, United States

**Keywords:** machine learning, microbiology, high throughput experiments, computational simulations, accelerate discovery

In recent years, Machine Learning (ML) has impacted various fields, not just business and social sciences, but also basic sciences. Those of us who became scientists before ML became so popular, have witnessed with amazement how its impact has grown and how useful the incorporation of ML techniques has been for our own research. Despite this, we have noticed that the impact of ML on microbiology has lagged. While experiments in microbiology now produce large amounts of data, and modeling techniques build bridges with experiments, the impact of ML has been slower to materialize.

Motivated by all of this, we decided to assemble an editorial team of theorists, experimentalists, and computer scientists to invite papers that combine these three areas in microbiology. The result is the Research Topic you are reading, which has also been made possible by the insights of the Frontiers in Microbiology editorial team. This Research Topic contains four papers that cover a variety of areas and applications of ML in microbiology.

The manuscript by Bommanapally et al. describe a ML-based image super-resolution (SR) technique for improving the image quality of microscopy images that will improve the synthesis of high-quality microscopy images. Muralidharan et al. assess how training ML taxonomic classifiers with computationally-generated data can significantly skew the assignment of microbial community sequence annotations and provide guidance toward more resilient ML classifiers.

Yang et al. propose a novel ML framework to address the challenge of predicting synthetic promoter strength in metabolic engineering and synthetic biology. Applied to the Trc synthetic promoter library, the new approach significantly improved model performance by achieving up to a 61.30% enhancement. The study conducted by Huang et al. evaluates the effectiveness of various machine learning methods and descriptors in predicting psychrophilic enzymes. The results could aid in the design and identification of cold-active enzymes.

Overall, these manuscripts showcase the potential of using machine learning in microbial studies, and we hope the scientific community will greatly benefit from their insights.

## Author contributions

MF-C: Writing – original draft, Writing – review & editing. PZ: Writing – original draft, Writing – review & editing. BL: Writing – original draft, Writing – review & editing. JW: Writing – original draft, Writing – review & editing. AB: Writing – original draft, Writing – review & editing. AD: Writing – original draft, Writing – review & editing.

